# The Possible Importance of β3 Integrins for Leukemogenesis and Chemoresistance in Acute Myeloid Leukemia

**DOI:** 10.3390/ijms19010251

**Published:** 2018-01-15

**Authors:** Silje Johansen, Annette K. Brenner, Sushma Bartaula-Brevik, Håkon Reikvam, Øystein Bruserud

**Affiliations:** 1 Section for Hematology, Department of Medicine, Haukeland University Hospital, N-5021 Bergen, Norway; silje.johansen@helse-bergen.no (S.J.); annette.brenner@k2.uib.no (A.K.B.); hakon.reikvam@k2.uib.no (H.R.); 2 Section for Hematology, Institute of Clinical Science, University of Bergen, 5007 Bergen, Norway; sushma.bartaula@iko.uib.no

**Keywords:** acute myeloid leukemia, integrin, beta3

## Abstract

Acute myeloid leukemia (AML) is an aggressive bone marrow malignancy where the immature leukemia cells communicate with neighboring cells through constitutive cytokine release and through their cell surface adhesion molecules. The primary AML cells express various integrins. These heterodimeric molecules containing an α and a β chain are cell surface molecules that bind extracellular matrix molecules, cell surface molecules and soluble mediators. The β3 integrin (ITGB3) chain can form heterodimers only with the two α chains αIIb and αV. These integrins are among the most promiscuous and bind to a large number of ligands, including extracellular matrix molecules, cell surface molecules and soluble mediators. Recent studies suggest that the two β3 integrins are important for leukemogenesis and chemosensitivity in human AML. Firstly, αIIb and β3 are both important for adhesion of AML cells to vitronectin and fibronectin. Secondly, β3 is important for the development of murine AML and also for the homing and maintenance of the proliferation for xenografted primary human AML cells, and for maintaining a stem cell transcriptional program. These last effects seem to be mediated through Syk kinase. The β3 expression seems to be regulated by HomeboxA9 (HoxA9) and HoxA10, and the increased β3 expression then activates spleen tyrosine kinase (Syk) and thereby contributes to cytokine hypersensitivity and activation of β2 integrins. Finally, high integrin αV/β3 expression is associated with an adverse prognosis in AML and decreased sensitivity to the kinase inhibitor sorafenib; this integrin can also be essential for osteopontin-induced sorafenib resistance in AML. In the present article, we review the experimental and clinical evidence for a role of β3 integrins for leukemogenesis and chemosensitivity in AML.

## 1. Introduction

Acute myeloid leukemia (AML) is an aggressive malignancy characterized by the bone marrow infiltration of immature leukemia cells [1,2]. The median age at the time of first diagnosis is 65–70 years, and the only possibility for cure is intensive chemotherapy that may be followed by allogeneic stem cell transplantation [3]. However, even for patients receiving intensive treatment, there is a relatively high risk of chemoresistant relapse, and this treatment is not possible for unfit and elderly patients due to the unacceptable risk of treatment-related mortality. The acute promyelocytic leukemia (APL) variant is an exception, and has a much better prognosis. In this review, the term AML refers to the non-APL variants; for these patients, there is a need for new therapeutic strategies both for the younger and elderly patients.

The bone marrow stromal elements, i.e., extracellular molecules and nonleukemic stromal cells, support leukemogenesis in human AML [4,5,6,7]. The integrins are one of the major families of cell adhesion receptors [8], and may be important for these AML-supporting effects. In the present review, we focus on the β3 integrin subset and their possible role in human AML.

## 2. The Integrin Family

**Figure 1:**
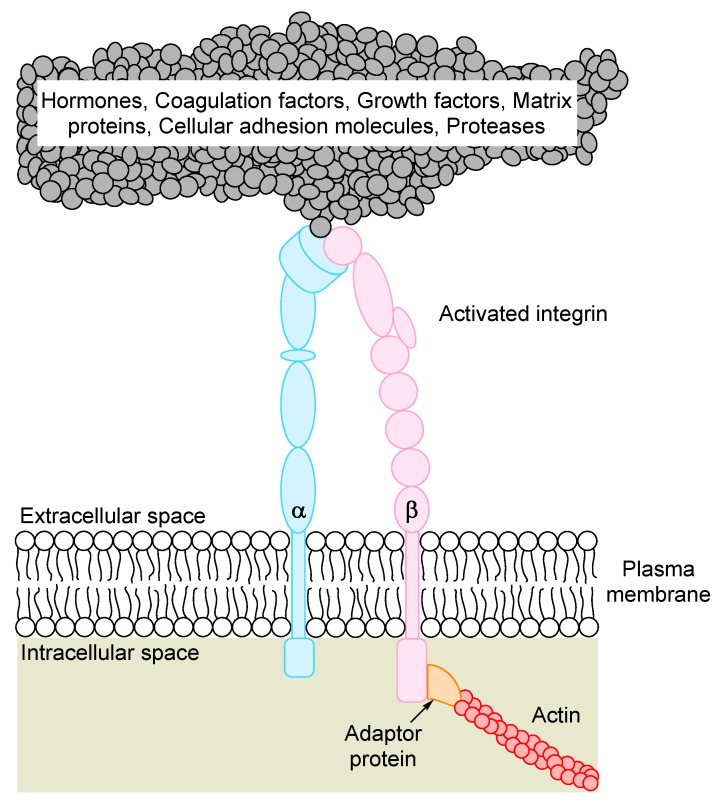
The integrin heterodimer consisting of one α and one β chain. In its activated state, the heterodimer interacts with the extracellular matrix through binding to large structural proteins such as collagen or fibrinogen, or with receptors on neighboring cells. The short cytoplasmic tail interacts with a vast variety of ligands, with members of the cytoskeleton comprising the largest subgroup. The main classification of ligands is shown at the top of the figure (for details see Table 1).

The integrins are non-covalently linked heterodimers containing an α and a β chain, and the dimers function as receptors for extracellular stromal molecules or cell surface molecules (Figure 1) [8,9,10,11,12]. Both subunits are transmembrane proteins containing large extracellular domains, a single transmembrane domain and a small cytoplasmic tail. Mammalian genomes contain 18 α and 8 β subunits, and 24 αβ-dimers have been identified at the protein level. Integrins coordinate the assembly of both cytoskeletal polymers and intracellular signaling complexes, and, on the cell surface, the integrins bind to extracellular matrix molecules or counter-receptors on neighboring cells. These linkages thereby integrate cells with their microenvironment, e.g., AML cells with the extracellular matrix or neighboring nonleukemic cells.

Adhesion mediated by integrins comprises a diverse family of cellular contacts essential for the organization of individual cells into tissues. The integrin adhesome includes 232 scaffold, cytoskeletal and signaling proteins [41]; 148 of these molecules are referred to as intrinsic proteins that reside within the adhesion site, whereas the remaining 84 components associate transiently with the integrins. A classification of the involved molecules is given in Table 2; it can be seen that the downstream signaling from integrins involves a large number of intracellular molecules with various functions. Several of these molecules can also be involved in carcinogenesis/leukemogenesis, including the guanosine triphosphatase (GTPase) activating proteins and the GTPases.

## 3. The β3 Integrin (ITGB3) Associations with Clinico-Pathological Features in AML

### 3.1. Regulation of ITGB3 Expression in AML Cells; the Importance of Troponin, PI3K and Monocytic Differentiation

Experimental studies suggest that thrombopoietin increases the activation of αIIbβ3 integrin, and the adhesion of leukemic cells to fibrinogen is thereby increased [13]. This increased binding is caused by recognition of the Arg–Gly–Asp (RGD) sequence on the fibrinogen ligand. The effect is only seen for αIIbβ3, but not for αVβ3 integrins, and signaling through phosphatidylinositol-3-kinases (PI3K) is important for the increased integrin expression/binding. The number of receptors per AML cell seems to be comparable with the expression in carcinoma and endothelial cells, and the leukemia cell levels of both αV and β3 chains seem to increase further in response to induction of monocytic differentiation [42]. Finally, there seems to be a variation in the expression within AML cell populations and a two-fold variation of αIIbβ3 protein expression has been detected even within more homogeneous AML cell line populations [43].

### 3.2. Associations between ITGB3 Expression and Clinico-Pathological Features

A high expression of ITGB3 at the mRNA level is associated with higher age and chemoresistance, i.e., a higher fraction of these patients with high ITGB3 expression had cytogenetic abnormalities or Fms-like receptor tyrosine kinase 3 (FLT3)-internal tandem duplications (ITD) associated with adverse prognosis [44]. However, these patients had, in addition, lower peripheral blood blast counts, lower blast percentage in the bone marrow and higher peripheral blood platelet counts. Taken together, these observations suggest that high ITGB3 expression is part of a high-risk AML cell phenotype, also characterized by differences with regard to AML cell trafficking (i.e., lower peripheral blood blast count) as well as a different influence on the persisting normal hematopoiesis (i.e., higher levels of circulating platelets) compared with other patients.

αIIbβ3 dimers (i.e., CD41 and CD61) are regarded as markers of megakaryocytic differentiation, as these dimers are expressed by megakaryocytes and platelets [45,46]. This integrin seems to have an increased expression in the uncommon acute megakaryoblastic variant of AML. However, the functional importance of αIIbβ3 may not be limited to the megakaryoblastic variant because αIIb together with α5, β1 and β3 are all important for adhesion of the erythroleukemia cell line (HEL) to vitronectin and fibronectin [47].

## 4. The Promiscuity of β3 Integrin Ligand Binding

A characteristic of most integrins is their ability to bind a wide variety of ligands; at the same time, many matrix molecules and cell surface adhesion molecules can bind multiple integrin receptors [48]. A classification of the various integrins based on their binding characteristics has been described in detail in a previous review [9], and integrins can be classified into four subsets based on their ligand binding. Both β3 integrins belong to the subgroup of RGD binding integrins, i.e., integrins recognizing ligands containing an RGD tripeptide sequence. These integrins are among the most promiscuous and bind to a large number of ligands, including extracellular matrix and soluble vascular ligands [13,14,15,16,17,18,19,20,21,22,23,24,25,26,27,28,29,30,31,32,33,34,35,36,37,38,39,40]. Important ligands for the β3 integrins are given in Table 1. It can be seen from the table that many of these ligands are expressed in the bone marrow by various AML-supporting stromal cells, including cells in the stem cell niches. Many of these ligands are known to affect AML cells, but for several of them it is not known whether these effects are mediated through ligation of β3 integrins, other integrins or different receptors.

## 5. β3 Integrins and Spleen Tyrosine Kinase (SYK) Activation in Murine Models of AML

### 5.1. ITGB3 Shows High Expression in the MLL-AF9 Mouse Model of AML

**Figure 2:**
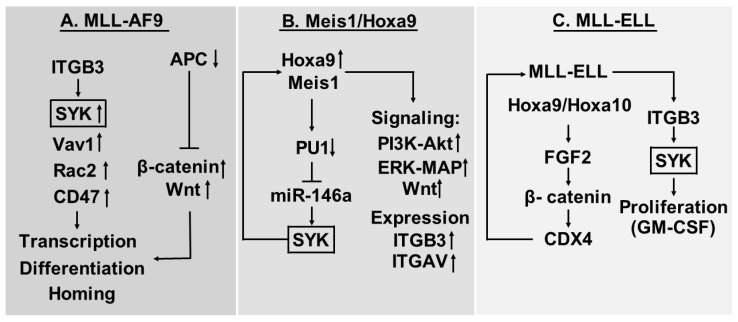
Animal models of AML—a summary of the MLL-AF9 (**A**), Meis1-HoxA9 (**B**) and MLL-ELL (**C**) murine models. For additional details and references, see the text.

Several MLL translocations transform committed hematopoietic progenitors; one of them, MLL-AF9, is the basis for the MLL-AF9 mouse model of AML (Figure 2A). This leukemia variant depends on Wnt-β-catenin; the negative β-catenin regulator Apc is decreased [49] and at the same time this leukemia is also characterized by high expression of ITGB3 as well as β-catenin (Ctnnb1) and High mobility group box 3 (Hmgb3) [50]. Studies of the intracellular signaling downstream to β3 integrins suggest that Syk, Vav1, Rac2 and CD47 are all activated. However, Syk activation seems to be of particular importance for the effects of ITGB3 on AML cell homing, transcriptional regulation in leukemic stem cells and differentiation induction of the leukemic cells.

As described above, this leukemia is based on an MLL-translocation and is dependent on Wnt-β-catenin signaling. However, several studies have demonstrated that Wnt-β-catenin is important also in AML with other genetic abnormalities, including del(5) [51], t(8;21) [52], normal karyotype AML [53], as well as FLT3-driven AML [52]. This pathway seems important for the survival of AML stem cells, and overexpression of this pathway has been associated with a poor prognosis in human AML [54]. Targeting of Wnt-β-catenin has also been suggested as a possible therapeutic strategy in human AML [55]. Thus, the Wnt-β-catenin pathway seems important for various subsets of AML and not only patients with MLL-translocations, and the possible role of ITGB3 suggested by the observations in this murine AML models may therefore be relevant also for several other AML subsets and not only this specific MLL-variant.

### 5.2. Myeloid Ectopic (Viral) Insertion Site-1 (Meis1)/Hoxa9 Driven AML Cells Depend on Meis1-Induced Syk Expression and ITGAV/ITGB3 Induced Syk Activation

The importance of Syk was also investigated in another murine AML model where the transcription factor Meis1 drives myeloid leukemogenesis in the context of HomeboxA9 (HoxA9) overexpression (Figure 2B) [56]. In this model Meis1, increased Syk overexpression through a Meis1 dependent feed-back loop: Meis1 acts through downregulation of transcription factor PU1 to increase the expression of mirR-146a that directly increases Syk expression. Syk signaling then induces Meis1 and thereby recapitulates leukemogenic features of the HoxA9/Meis1 driven leukemia. Syk inhibition with disruption of the Meis1/PU1/miR-146a/Syk loop has antileukemic effects and prolongs survival of these mice, even though activation of Wnt is also involved in the cell transformation, and Meis1 also enhances signaling through PI3K/Akt and Mitogen activated protein/extracellular signal-regulated kinase (Erk/MAP) kinase pathways in addition to Syk activation. Finally, additional studies showed that Meis1 increased the expression of both ITGB3 and integrin αV (ITGAV) and thereby upregulates the cell surface levels of this integrin heterodimer [56]. Knock-out studies suggested that the Syk activation was at least partly dependent on ITGB3 also in this model. Thus, a combined action of ITGB3 expression, Wnt signaling and Syk activation seems to be important both in MLL-AF9 and Meis1/HoxA9 driven disease.

Several studies suggest that Meis1/HoxA9 are also important in human AML. Overexpression of HoxA9 is associated with an adverse prognosis and high expression is associated with a variety of genetic abnormalities not only including MLL translocations, but also Nucleoporin98 (NUP98) fusion, Nucleophosmin 1 (NPM1) mutations isocitrate dehydrogenaseIDH mutations, CDX deregulation, MYST translocations, c-AMP response element-binding protein (CREBBP) involving abnormalities and monocytic leukemia zinc finger protein (MOZ) fusions [57,58,59,60]. Meis1/HoxA9 then seem to belong to a set of homeodomain transcription factors associated with adverse prognosis [58,61]. Taken together, these observations suggest that Meis1/HoxA9 are important for various subsets of human AML; the observations in this animal model of AML suggesting a role of β3 integrins in the pathogenesis and in the development of clinical chemoresistance, may therefore be relevant also for human AML.

### 5.3. Proliferation of AML Cells Expressing the MLL-ELL Fusion Protein is Increased by Cooperation between Fibroblast Growth Factor (FGF) 2 and ITGAV/ITGB3 Integrins

In another experimental murine AML model, the MLL-ELL fusion protein activates aberrant expression of HOX genes (Figure 2C) [38,62]. HoxA9 and HoxA10 then increase the expression of the FGF2 gene, and the autocrine stimulation by FGF2 stabilizes β-catenin that increases the expression of CDX4; this last mediator targets HoxA9 and HoxA10 and thereby augments the effect of the MLL-ELL fusion protein. Thus, the autocrine FGF2 stimulation is a part of a MLL-ELL initiated loop including MLL-ELL, HoxA9 and HoxA10/FGF2/β-catenin/CDX4 and then back to HoxA9 and HoxA10. The MLL-ELL fusion protein also increased ITGB3 expression and activation of the αVβ3 integrin that caused integrin-mediated Syk activation leading to increased proliferative responsiveness to Granulocyte macrophage colony stimulating factor (GM-CSF). Taken together, the observations in this experimental model also support a role of the αVβ3 integrin and its activation of Syk in leukemogenesis.

## 6. The Role of αVβ3 Integrins Human AML

### 6.1. HOX Genes and β-Catenin

The M9 cells are a human model of MLL-AML; these cells were derived from umbilical cord blood cells transduced with the MLL-ELL oncogene [56]. These cells express β3 integrins, and transplantation of these cells into immunocompromised mice showed that β3 integrins were important for later development of leukemia. Furthermore, ITGB3/ITGAV is also expressed by primary human AML cells both with and without MLL-rearrangement [56], and xenografting experiments suggest that the β3 integrins were then important for engraftment and thereby disease development both in patients with and without MLL rearrangements. These observations suggest that β3 integrins are important also for human disease development both in MLL and non-MLL variants of AML.

Previous studies of murine AML suggest that HoxA9/HoxA10 are involved in leukemogeneisis through their effects on the expression/activation of αVβ3 integrins and Syk (Figure 2B,C) [38]. Furthermore, studies of human AML suggest that HOX genes can be important in human leukemogenesis, and high HOX expression seems to identify a distinct subset of patients [63,64]. Similar to the murine AML models described above [38], HOX expression seems to be associated with increased expression of FGF2, αVβ3 integrins and CDX4 compared with normal hematopoietic cells. The AML patients showing the highest HOX expression also show the highest levels of FGF2, αVβ3 and CDX4, and this subset of patients with the highest expression are also characterized by a higher proliferative response to exogenous GM-CSF and a stronger antiproliferative effect of FGF2/Syk inhibition [38]. Thus, the HOX/FGF2/β-catenin/CDX4 and the HOX/αVβ3/Syk cooperation first identified in murine models of AML seems important also in human leukemogenesis at least for a subset of AML patients that mainly includes patients with intermediate cytogenetics [63].

The role of β-catenin was further investigated in a recent study [44] describing an association between ITGB3 expression and survival in human AML (see Figure 2A,B). This association was seen especially for patients with normal cytogenetics and FLT3-ITD. The authors also presented experimental evidence for a role of αVβ3 in osteopontin-induced chemoresistance in FLT3-ITD AML, and this seemed to be caused by activation of the αVβ3/PI3K/Akt/glycogen synthase kinase-3 β (GSK3-β)/β-catenin pathway. These observations further support that the cooperation between HOX/FGF2/β-catenin/CDX4, HOX/αVβ3/Syk and αVβ3/PI3K/Akt/GSK3-β/β-catenin is important for leukemogenesis and probably also chemosensitivity in human AML.

### 6.2. Modulation of Syk and Focal Adhesion Kinase (FAK) Activation by β3 Ligation

The two β3 integrins can bind a wide range of ligands, and the downstream activation of the nonreceptor protein tyrosine kinases Syk and FAK is in addition dependent on how the ligand is presented to the integrin [13]. This has been clearly demonstrated by fibrinogen; an experimental study of a megakaryoblastic leukemic cell line showed that soluble fibrinogen caused tyrosine-phosphorylation of Syk but a dephosphorylation of FAK whereas solid-phase fibrinogen caused immediate FAK phosphorylation followed by delayed Syk phosphorylation. A combined soluble and solid phase fibrinogen exposure caused tyrosine phosphorylation of the β3 and at the same time complex formation with Syk; the further translocation of Syk to the cytoskeleton seems to be a two-step process and one of these later steps is also β3 dependent [65].

The importance of FAK in human AML is summarized in Table 3. Several studies have described a role of FAK in the regulation of proliferation, chemoresistance and migration of human AML cells [66,67,68,69,70]. Even though these studies suggest that the functional interactions between upstream β3 integrins and FAK has a clinical relevance, additional studies are definitely needed to clarify the possible importance of β3 integrins initiated signaling for the role of FAK expression and activation in human AML. However, the observed association between FAK activation and β3 integrin expression suggest that these integrins contribute involved in FAK activation.

### 6.3. Clinical Evidence for a Role of β3 Integrins in Human AML; the Stories of SPARC and TRIM62

The secreted-protein-acidic-cysteine-rich (SPARC) encodes the matricellular protein osteonectin that has both intracellular and extracellular functions including regulation of the growth factor families transforming growth factor β (TGFβ), FGF, vascular endothelial growth factor (VEGF) and platelet-derived growth factor (PDGF); a recent study described that the overexpression of this protein independently predicted an adverse outcome in patients with normal karyotype AML [34]. However, its prognostic impact may depend on the biological or genetic context because AML with MLL rearrangements is also associated with an adverse prognosis, but shows downregulated SPARC, and increased levels have been described for the favorable AML variants with t(8;21) and inv(16) (for references see [34]). Experimental studies suggested that the increased SPARC expression was mediated by SP1/NFκB/miR-29b, and the secreted SPARC activated the integrin-linked kinase-Akt-GSK3β pathway. GSK3β will induce degradation of β-catenin, and GSK3β inactivation through the Akt-mediated phosphorylation will thereby increase β-catenin levels. Thus, these observations also support the hypothesis that integrin/β-catenin signaling is important for leukemogenesis and/or chemosensitivity at least in subsets of AML patients.

Tripartite motif-62 (TRIM62) is a putative tumor suppressor gene, and low expression of this mediator has been associated with adverse prognosis and shorter remission duration, event-free survival and overall survival in patients with intermediate-risk cytogenetics [71]. In this study, age and TRIM62 levels were the most powerful independent prognostic factors. However, among the proteins that were most strongly downregulated in patients with low TRIM62 (i.e., adverse prognosis) were both the integrin-β3 dimers (associated with adverse prognosis previously [44]) and their ligand fibronectin.

Taken together these two studies suggest that β3 integrin expression has a prognostic impact in AML patients receiving intensive therapy, but this impact seems to differ between patient subsets.

## 7. β3-Integrins, Intracellular Signaling and Transcriptional Regulation—A Summary of Our Current Evidence

The present evidence from animal models as well as human studies described above suggest that β3-integrin initiates downstream signaling that alters transcriptional regulation. Several pathways seem to be involved, and the most important observations are summarized below:

1. The extracellular SPARC molecule may be important for regulation of cytokine responses (e.g., FGF2 and possibly GM-CSF responses) and thereby interact with the functional effects of β3-integrin signaling.
2. Several cell surface molecules seem to be important for the signaling, including CD47 and β-catenin; this last protein has a dual function and is important both for cell adhesion and transcriptional regulation. Another cell surface proteins being important for the signaling is CD47.
3. Syk seems to be an important downstream mediator, possibly the most important.
4. However, several pathways seem to be involved, including Wnt-signaling, PI3K-Akt, and Erk-MAP. The importance of Vav1 (guanine nucleotide exchange factor) and Rac2 (a GTPase) in the MLL-AF9 model also suggest that G-protein dependent signaling may be involved.
5. Several transcriptional regulators also seem to be involved, including β-catenin, Meis1, miR-146a, PU1, HoxA9, HoxA10 and CDX4.

Thus, several cell surface molecules, intracellular pathways and transcriptional regulators are important for the function of β3 integrins in human AML. The contribution of each molecule/mediator seems to depend on the biological context/experimental model, and this last observation suggests that their importance may also differ between biologically heterogeneous AML patients.

## 8. The Soluble Isoform of β3 Integrins

Alternative splicing is an important mechanism to increase the functional diversity of integrins, and a soluble β3 (sβ3) variant has been detected in the serum for a subset of AML patients [72]. This variant represents an alternatively spliced and truncated variant. Additional studies showed that this sβ3 integrin increased natural killer (NK) cell proliferation, interleukin-2 (IL2)-induced NK cell release of interferon-γ (IFN-γ) and tumor necrosis factor-α (TNF-α), NK cell expression of granzyme B and Fas ligand and NK cell cytotoxicity. This included increased cytotoxicity against AML cells that was probably caused by a combined effect of the cytokine release, Fas-induced apoptosis and increased cytotoxic capacity.

Antileukemic immune reactivity is especially important in AML patients treated with allogeneic stem cell transplantation, and the NK cells seem to contribute to this reactivity [73]. The capacity of primary AML cells to release sβ3 integrins may therefore be important for the posttransplant antileukemic effects mediated by the graft or donor NK cells, and it may also contribute to patient heterogeneity with regard to the efficiency of the posttransplant antileukemic immune reactivity.

## 9. The Possible Importance of Cooperation between Different Integrins

Experimental studies of normal leukocytes suggest that the function of one integrin can be regulated by another integrin. Several examples are known: α5β2 ligation activates α2β1 integrins in monocytes and β3 ligation can activate the function of αMβ2 integrin. However, αVβ3 can also inhibit the function of α5β1 in the human AML cell line K562 [74,75,76] and this last αVβ3 effect does not seem to be a direct interaction between the two types of integrins but rather a crosstalk at the intracellular signaling level. Most studies of integrins in human AML have focused on one or only a few integrins, and future studies should probably further address the question whether the integrin profile is more important than single integrin expression. Previous studies suggest that β1 integrins are important in human AML, and it will therefore be particularly important to investigate the possible crosstalk between β3 and β1 integrins in human AML [77,78,79,80].

## 10. Summarizing and Concluding Comments

Several integrins are expressed by primary human AML cells, and previous studies have shown that they can be used together with other molecules as markers for leukemia cell differentiation and thereby as markers for identification of patient subsets. More recent studies have lately shown that at least certain integrins or their downstream mediators are potential therapeutic targets in AML.

The integrin family consists of 24 heterodimers that can bind to a wide range of ligands expressed on the surface of neighboring cells or by extracellular matrix molecules. Many of them are expressed by primary human AML cells, including the two β3 integrins αIIbβ3 and αVβ3 integrins. β3 integrins and especially αVβ3 seem to be important for disease development and chemosensitivity in human AML at least for certain subsets of patients, including patients with MLL translocations. Furthermore, the integrin interactome consists of a large number of intracellular mediators, but especially β-catenin, the Syk kinase and several HoxA genes seem to be important together with the FGF2 receptor for the downstream signaling of β3 integrins in human AML cells. Thus, Syk inhibition as well as β3-specific antibody blocking may be a possible strategy for inhibition of AML-supporting signaling circuits.

The β3 integrins show downstream crosstalk with other integrins as well as other intracellular signaling pathways. This includes both PI3K-Akt and NFκB signaling as well as certain β1 integrins. Thus, combination of β3-inhibition and other targeted therapies may become possible. There may also be a crosstalk with the chemokine system and intracellular signaling initiated by chemokine receptors [70,78].

Integrins seem to be important for the development of chemoresistance in human AML. Firstly, galectin 1 seems to induce sorafenib-resistance in hepatocellular carcinoma through induction of αvβ3 integrin expression and activation of PI3K-Akt signaling [81]. Galectin-1 is also expressed in human AML bone marrow both by the leukemic cells and by stromal cells in the stem cell niches [82,83]. A recent study could not find any association between galectin expression in primary human AML cells and prognosis after conventional chemotherapy, but further studies are needed to clarify whether galectin-1 dependent chemoresistance is important for subsets of patients or for kinase inhibitory strategies. Secondly, α3 integrins as well as expression of the downstream FAK seem to be important for development of sorafenib resistance in hepatocellular carcinomas and mantle cell lymphoma [84,85]; αvβ3 integrin may then influence these mechanisms through the crosstalk between various integrin heterodimers (see above).

The possibility of using inhibition of β3 integrins in future AML treatment should thus be considered. However, a further characterization especially of patient heterogeneity with regard to integrin expression profile and integrin crosstalk is needed to clarify whether this therapeutic strategy will be effective only in certain patients. The β3 integrins are very promiscuous with regard to binding of various ligands (Table 1), and future studies have to address whether the binding of certain ligands is especially important in AML. Finally, it will be necessary to further investigate whether this therapeutic approach can be combined with conventional cytotoxic therapy or other targeted therapies before an optimal design of future clinical studies will be possible.

## Figures and Tables

**Table 1:** Classification of β3 integrin ligands and an overview of the importance ligands, their important functions and their integrin binding; for additional details see [9] and the Gene database [13,14,15,16,17,18,19,20,21,22,23,24,25,26,27,28,29,30,31,32,33,34,35,36,37,38,39,40].

Ligand	Integri Binding	Function of the Ligand in Human Acute Myeloid Leukemia (AML)	Key References
ADAM family members	αVβ3	ADAMTS-13, see von Willebrand factor (vWF) below.	
Androgens	αVβ3	A recent study described improved survival of elderly patients when androgens maintenance treatment was combined with intensive chemotherapy.	[14]
BSP	αVβ3	Bone sialo protein (BSP). No known effect in AML.	
Collagen	α10β3	Collagen IV promotes the migration and adhesion of primary human AML cells, MMP-9 is also increased. Collagen and collagen IV is present in human bone marrow. It is not known whether binding to integrins contributes to these effects or whether other receptors are responsible (e.g., the diskoid domain receptor 1).	[15]
COMP	αVβ3	Cartilage oligomeric matrix protein(COMP) This mediator is synthesized by osteoblasts and may thus be expressed in the bone marrow niches.	[16]
Connective tissue growth factor	αVβ3, αIIbβ3	Connective tissue growth factor (CTGF) is expressed in bone marrow stromal cells; it is regarded as a regulator of adipocyte differentiation and may influence leukemogenesis both through direct effects on the AML cells and through indirect effects on AML-supporting stromal cells. AML cells induce its expression in bone marrow mesenchymal cells.	[17,18]
Cyr61	αIIbβ3, αVβ3	Cystein-rich 61(Cyr61) is released by stromal cells, it is released as a matricellular protein and it increases the proapoptotic effects of mitoxantrone in AML-stromal cell cocultures.	[19]
Del-1	αVβ3	The secreted glycoprotein Developmental endothelial locus-1 (Del-1) is expressed endothelial cell, becomes associated with extracellular matrix or cell surfaces and regulates hematopoiesis in the bone marrow stem cell niche.	[20]
Fibrillin	αVβ3	Murine studies have demonstrated that the extracellular matrix protein, fibrillin, is expressed in the bone marrow and functions as a regulator of normal hematopoiesis.	[21]
Fibrinogen	αIIbβ3, αVβ3	The plasma fibrinogen levels at the time of diagnosis seem to have a prognostic impact and are associated with an adverse outcome in AML patients. This impact is not caused by increased early mortality, but it is not known whether this long-term effect is caused by a direct effect of fibrinogen on the AML cells. Both soluble and solid-phase fibrinogen induces Syk signaling in human megakaryoblastic cell lines.	[13,22]
Fibronectin	αIIbβ3, αVβ3	Experimental studies suggest that AML cell adhesion to fibronectin increase leukemia cell proliferation, accelerate S-phase entry and cause accumulation of the cell cycle inhibitor CDC25A. This CDC25A accumulation was caused by decreased degradation. Activation of PI3K-Akt-mTOR seemed to be important for this adhesion-dependent growth enhancement. Fibronectin adhesion inhibited the proliferation of normal CD34^+^ bone hematopoietic cells.	[23]
ICAM-4	αVβ3, αIIbβ3	Intercellular adhesion molecule-4 (ICAM-4) is expressed by erythroid cells and seems important in erythropoiesis, but it is not known whether it is important in AML.	[24]
L1	αVβ3, αIIbβ3	L1 is expressed by human monocytes and may thus be expressed in the bone marrow stem cell niches.	[25]
MFG-E8	αVβ3, αVβ5	The Milk fat globule-EGF-factor 8 protein (MFG-E8) is expressed and released by bone marrow macrophages and is thus present in the AML cell microenvironment.	[26]
MMP-2	αVβ3	Matrix metalloprotease 2 (MMP-2) is constitutively released by primary human AML cells for most patients and is involved in AML cell migration; it may even be important for the extracellular migration of leukemic cells. An adverse prognostic impact of constitutive MMP-2 release has been suggested.	[27,28,29]
Osteopontin	αVβ3	Monocytic differentiation in human AML cells seems to be associated with increased expression of both ITGαV and osteopontin. High osteopontin serum levels seem to be associated with an adverse prognosis in human AML, but this impact differs among patients and is most clearly seen for patients with intermediate risk factors.	[30,31,32]
PCAM	αVβ3	Mesenchymal stem cells express Platelet cell adhesion molecule (PCAM); this ligand is thus expressed in the bone marrow stem cell niches where leukemic stem cells locate.	[33]
SPARC	αVβ3?	Secreted-Protein-Acidic-Cysteine Rich (SPARC) Seems to induce β3-catenin signaling at least in subsets of human AML.	[34]
Thyroid hormones	αVβ3	A matched case-control study with 28 children/patients with AML showed that extreme Thyroid stimulating hormone (TSH) levels, both high and low at neonatal screening, were associated with decreased risk of AML	[35]
Trombospondin	αVβ3, αIIbβ3	Thrombospondin induces apoptosis in AML cell lines and also in primary human AML cells, but this effect may be due to ligation of CD36. The effect is antagonized by thrombopoietin, a mediator that is often increased in AML patients receiving intensive chemotherapy.	[36,37]
Vitronectin	αIIbβ3, αVβ3	Adhesion of Mixed lineage leukemia-Eleven-nineteen lysine-rich leukemia (MLL-ELL) murine myeloid progenitor cells to vitronectin activates/phosphorylates β3 integrins and Syk kinase.	[38]
vWf	αVβ3, αIIbβ3	ADAMTS-13 is essential for maintaining the keeping normal sized of the vWF; it cleaves the multimer into smaller forms. Low plasma levels of ADAMTS-13 seems to be associated with an adverse outcome in human AML, but it is not known whether this is due to an effect of ADAMTS-13/vWF directly on the AML cells or whether it represents a secondary reactive mechanism.	[39,40]

**Table 2:** The integrin adhesome.

Actin and Actin Regulators (18 Members)
Closely related to the cytoskeleton
Adaptor proteins contain a variety of protein-binding modules that link protein-binding partners
together and facilitate formation of larger complexes.
The integrins are the largest subset of proteins in this group
Channel proteins (5 members)
Chaperones (3 members)
E3 ligases
GTPase activating proteins (14 members)
Guanine nucleotide exchange factor (16 members)
GTPases (6 members)
Proteases (4 members)
Phosphatidyl inositol (PtdIns) kinases (2 members)
PtdIns phosphatases (3 members)
RNA or DNA regulation (4 members)
Serine/Threonine (Ser/Thr) kinases (10 members)
Ser/Thr phosphatases (5 members)
Tyrosine (Tyr) kinases (10 members)
Tyr phosphatases

**Table 3:** The role of the non-receptor tyrosine kinase, focal adhesion kinase (FAK), in human AML.

FAK expression is significantly higher in MDS that later transform to AML [66]. FAK expression is detected only for a subset of patients; FAK was then activated at Tyr-397 and this was associated with increased blast migration, increased marrow cellularity and adverse prognosis [67]. High FAK expression is associated with unfavorable cytogenetics and an increased risk of AML relapse, and this expression correlates with integrin β3 expression [68]. FAK splice variants are abnormally expressed in leukemic stem cells from patients with adverse prognosis, and these abnormal variants cause activation of β-catenin and thus replace the Wnt-controlled β-catenin activation [66]. Constitutive activation of FAK activation has an essential role in nuclear translocation of Signal transducer and activator of transcription (STAT5) in AML with FLT3 and KIT mutations [69]. Integrin α4β1 expression, CXC chemokine receptor 4 (CXCR4) expression and FAK activation seem to have additive adverse prognostic impacts [70].

## Abbervations

AML	Acute myelogen leukemia
APL	Acute promyelocytic leukemia
COMP	Cartilage oligomeric matrix protein
CREBBP	CREB binding protein
CTGF	Connective tissue growth factor
Cyr61	Cystein-rich61
Ctnnb1	β–catenin
CXCR4	CXC chemokine receptor 4
Del-1	Developmental endothelial locus-1
ELL	Eleven-nineteen lysine-rich leukemia
FAK	Focal adhesion kinase
FLT3	Fms-like receptor tyrosin kinase 3
GSK3-β	Glycogen synthase kinase-3 β
GTPase	Guanosine triphosphatase
GM-CSF	Granulocyte-macrophage colony-stimulatingfactor
HEL	Erythroleukemia cell line
HoxA9	Homebox A9
Hmgb3	High mobility group box 3
ICAM-4	Intercellular adhesion molecule-4
IL2	Interleukin-2
IFN-γ	Interferon-γ
ITD	Internal tandem duplications
ITGAV	Integrin αV
ITGB3	Beta3 intergrin
MAP/ERK	Mitogen activated protein/extracellular signal-regulated kinase
MEIS1	Myeloid ectopic (viral) insertion site-1
MFG-E8	Milk fat globule-EGF-factor 8 protein
MLL	Mixed lineage leukemia
MMP-2	Matrixmetalloprotease 2
MOZ	Monocytic leukaemia zinc finger protein
NK	Natural killer (cell)
NPM1	Nucleophosmin 1
NUP98	Nucleoporin98
PDGF	Platelet derived growth factor
PCAM	Platelet cell adhesion molecule
PtdIns	Phosphatidyl inositol
PI3K	Phosphatidylinositol-3-kinases
RGD	Arg-Gly-Asp
Ser/Thr	Serine/Threonine
S β3	Soluble β3
SPARC	Secreted-Protein-Acidic-Cysteine Rich
STAT5	Signal transducer and activator of transcription-5
SYK	Spleen tyrosin kinase
TGF-β	Transforming growth factor-β
TNF-α	Tumor necrosis factor-α
TRIM62	Tripartite motif-62
TSH	Thyroid stimulating hormon
Tyr	Tyrosine
VEGF	Vascular endothelial growth factor
vWF	Von Willebrand Factor
